# Genome-Wide Detection of Selection Signatures for Pelt Quality Traits and Coat Color Using Whole-Genome Sequencing Data in American Mink

**DOI:** 10.3390/genes13111939

**Published:** 2022-10-25

**Authors:** Shafagh Valipour, Karim Karimi, Duy Ngoc Do, David Barrett, Mehdi Sargolzaei, Graham Plastow, Zhiquan Wang, Younes Miar

**Affiliations:** 1 Department of Animal Science and Aquaculture, Dalhousie University, Truro, NS B2N 5E3, Canada; 2 Department of Pathobiology, University of Guelph, Guelph, ON N1G 2W1, Canada; 3 Select Sires Inc., Plain City, OH 43064, USA; 4 Livestock Gentec, Department of Agricultural, Food and Nutritional Science, University of Alberta, Edmonton, AB T6G 2H1, Canada

**Keywords:** American mink, pelt quality, selection signatures, pelt size, whole genome sequencing

## Abstract

Domestication and selection are the major driving forces responsible for creating genetic variability in farmed species. American mink has been under selection for more than 100 years for improved body size and pelt quality. This study aimed to identify the genomic regions subjected to selection for pelt quality traits, and coat color using the whole genome sequences of 100 mink raised in the Canadian Centre for Fur Animal Research (CCFAR) at Dalhousie Agriculture Campus (Truro, NS, Canada), and Millbank fur farm (Rockwood, ON, Canada). Measurements of three dried pelt characteristics (including pelt size (*n* = 35), overall quality of fur (*n* = 27), and nap size (*n* = 29)), and three coat color of Black, Stardust, and Pastel (Stardust_ Black (*n* = 38), and Pastel_Black (*n* = 41)) were used to assign animals to pairwise groups. Signatures of selection were detected using integrated measurement of fixation index (Fst), extended haplotype homozygosity (XP-EHH), and nucleotide diversity (θπ) tests. In total, overlapping top 1% of Fst and XP-EHH harbored 376 genes for pelt quality traits (110 for nap size, 163 for overall quality of fur, and 98 pelt size), and 194 genes for coat color (123 for Pastel_Black and 71 for Stardust_Black) were detected in different groups. Integrating results of Fst, and XP-EHH with the θπ test supported 19 strongly selected regions on chromosomes 3, 4, 5, 6, 7, 8, 9, and 10 that contained 33 candidate genes related to fur quality, hair follicle function, and pelt size traits. Gene ontology revealed numerous genes related to the hair cycle process and molting cycle process, epidermis development, Wnt signaling pathway and muscle development. This study provided the first map of putative selection signals related to pelt quality and coat color in American mink, which could be used as a reference for future studies attempting to identify genes associated with economically important traits in mink.

## 1. Introduction

The American mink (*Neogale vison*) is a semi-aquatic species of the carnivorous order native to north America and is the most important fur-bearing species used in the fur industry worldwide. The American mink was initially bred in captivity in 1866 in Canada [1]. Since then, due to its importance for fur industries, mink farming was extensively practiced in North America, Europe, and Asia. Farmed mink were selectively bred for improved litter size, pelt quality, disease resistance, body growth, and behavioral traits [2]. Evidence indicates that artificial selection during the last 150 years has driven the differentiation between farmed mink and wild populations [3,4,5]. Pelt quality and coat color are important breeding objectives because of their effects on the final economic value of fur. Short-haired large pelts with dense hair coverage and healthy guard hair hold the highest economic value in the fur industry. Black mink coats are the most used color in fur fashion industry as it can be worn with all other colors of clothing, and colors such as pastel and stardust are sold at high price because of their unique natural color that can meet consumer preferences for natural products [6,7].

In mink, a few attempts have been made to pinpoint genes associated with pelt quality traits, using genome-wide association studies (GWAS) [8] or linkage mapping [9]. However, only a limited number of significant quantitative trait loci (QTL) with small effects on their genetic variations have been identified [8,9]. Similarly, a few candidate genes potentially involved in pigmentation have been identified in mink, such as the *MLPH*, *LYST*, *TYR*, *MITF* and *TYRP1* genes [10,11,12,13,14]. In mice, more than 170 genes involved in pelage pigmentation have been detected [15]. Therefore, understanding molecular genetic mechanisms underlying pelt quality and coat color regulation in mink required further investigation.

The wild mink is originally dark brown, which is commonly known as standard dark brown or black mink [16,17,18]. Appearance of mutant colors was documented as early as 1929 in ranch-raised mink [18]. It has been suggested that restriction of free mating and increased inbreeding during high-intensity artificial selection in commercial farms led to increased homozygosity of natural recessive coat color mutations that have been accumulated in genome of individuals have led to the appearance of mutant color-types [19,20]. For instance, the appearance of Black Crystal, Himalayan coat colors and intensification of expressivity of the white piebald after multiple generations of selection for tame behavior have been reported in farmed mink [19,20]. During the last century, due to the economic profit offered by producing mutant coat colors, mink breeders have selectively bred mink for different coat colors which led to the creation of a great diversity of color-types in farmed mink. From the view of population genetics, the effect of artificial selection for pelt quality and coat color would leave detectable selection signatures within the mink genome [21,22]. Therefore, identifying the selection signatures underlying these traits would provide an opportunity to characterize the genomic regions contributing to the pelt quality and coat color traits in domesticated mink.

Availability of whole-genome sequencing (WGS) made it possible to discover sequence variants such as single nucleotide polymorphism (SNP) at a population scale that can facilitate the mapping of selection signatures at higher resolution than SNP microarrays or genotyping-by-sequencing data [23]. Multiple approaches have been developed to detect the patterns of selection signatures in the genome, based on different features of selective sweeps [24]. For instance, nucleotide diversity (θπ), which is the average number of pairwise nucleotide differences between sequences in a sample, cross population extended haplotype homozygosity (XP-EHH) that is based on the extend of linkage disequilibrium within the populations, and fixation index (Fst), which is the measure of real allele frequency differences between individuals in a population [22]. Selection signatures can be identified by the changes in the allele frequency spectrum, increase in homozygous genotypes, and extended linkage disequilibrium levels, i.e., long haplotypes exist with high frequency [22]. Therefore, using a combination of multiple statistics to detect the targets of selection is often a good option. 

Scanning genome for evidence of selection signatures has been extensively used for identification of genes and genomic regions related to disease phenotypes in human [25], or economic traits in crops [26], and livestock species [27]. In American mink, selection signature approaches were used to reveal the putative regions for response to Aleutian mink disease virus infection [28]. However, to our knowledge, no study investigated the selection signatures for pelt quality, pelt size and coat color traits in farmed mink. Therefore, the objectives of this study were to, (1) identify the selection signatures for pelt quality and coat color traits in American mink genome using WGS data, and (2) identify the candidate genes related to pelt quality, pelt size, and coat color traits. 

## 2. Material and Methods

### 2.1. Animals and Sampling

Phenotypic records were collected from animals born and raised in 2018 at the Canadian Center for Fur Animal Research (CCFAR) at Dalhousie University (Truro, NS, Canada) and Millbank Fur Farm (Rockwood, ON, Canada). Animal management and sampling procedures were performed in accordance with the standards of the Canadian Council on Animal Care [29] after approval by the Dalhousie University Animal Care and Use Committee (certification#: 2018-009). In December 2018, mink were euthanized using the approved method of carbon monoxide gas to provide a quick and humane death. Tongue tissues were collected from 100 animals for DNA isolation.

### 2.2. Animal Grouping

Mink used in this study were euthanized in December of 2018 and were shipped to the custom pelting facilities (Arcadia, NS, Canada) for pelting. Dried pelts were shipped to the North American Fur Auction (NAFA) house (Toronto, ON, Canada) where the evaluation of dried pelts was performed by certified technicians. Three pelt quality traits, including nap size, overall quality of fur, and pelt size; and three color-types, including black, stardust, and pastel, were used to divide animals into subgroups based on their phenotypic information. Nap size is defined as the length of guard hair protruding out of underfur [30]. It was scored into eight categories: ranging from extra short nap (category 1) to medium-long nap (category 8) [31]. For genomic analysis, we grouped the animals with extra short nap and short nap into subgroups of short nap (*n* = 14) and animals with medium nap to medium-long nap and medium-long nap into the long nap subgroup (*n* = 15). The overall quality of fur describes the general appearance of fur in terms of density of underfur, healthy appearance of guard hairs and underfur, and smooth and silky textures of fur. For overall quality of fur, animals were classified into high fur quality (*n* = 16), i.e., pelt of very high quality, fully prime, dense, and resilient underfur with good and even guard hair coverage and super silky textures, and low fur quality (*n* = 11), i.e., weakest pelts in terms of underfur, guard hair, uneven coverage of guard hairs and underfur, coarser guard hair, and weak general appearance. Pelt size is the length of dried pelt measured from the tip of nose to the base of tail. Since pelt size is influenced with sex, we only used pelts from female mink. Male pelts size was not included in this study as our sequenced population did not contain a sufficient number of males with small pelt size. Therefore, female pelts larger than 77 cm were assigned to large pelt size subgroup (*n* = 10) and pelts smaller than 59 cm were categorized as small pelt size (*n* = 25). In addition, since black or dark color is considered the mink wild color type for mink [16], we also examined the signatures of selection for coat color by comparing the stardust (*n* = 7) and pastel (*n* = 10) color-types versus black color-type (*n* = 31).

### 2.3. Whole-Genome Sequencing, Reads Alignment and Variant Calling

DNA was isolated from tongue samples using the Qiagen DNeasy Blood and Tissue Kit (Qiagen, Hilden, Germany). Sequencing and generation of paired-end libraries (100 bp pair-end reads) was performed using the BGISEQ-500 platform at Beijing Genomics Institute (BGI, Guangdong, China). After sequencing, sequencing adapters and low-quality reads were removed using SOAPnuke software 2.1.6 [32]. The filtered reads were aligned against the recent American mink reference genome (https://www.ncbi.nlm.nih.gov/assembly/GCF_020171115.1/; accessed on 15 September 2022) using Burrows-Wheeler Aligner (BWA) 0.7.17 [33]. The aligned files were converted to binary alignment map (BAM) format and sorted using SAMtools 1.11 [34] and the potential PCR duplicates were then removed using the MarkDuplicates command tool of Picard 2.26.8 [35]. The BAM files were then indexed by SAMtools 1.11. Finally, variant calling was performed with SAMtools 1.11 and Genome Analysis Toolkit 4.1.9.0 (GATK) pipeline using haplotypecaller 2.0.3 [36]. Quality control of variants was performed using VCFtools 0.1.16 [37]. Variants with minor allele frequency (MAF) < 0.05; maximum missing rate <1.0; deviating from Hardy–Weinberg equilibrium (*p*  <  10^−6^) were removed. Moreover, only bi-allelic variants on autosomal chromosomes were kept. After quality control, 9,922,758 bi-allelic variants from 100 individuals remained for further analysis. 

### 2.4. Detection of Selection Signatures

#### Pairwise Fixation Index (Fst)

The Fst values were calculated for each SNP according to Weir and Cockerham [38] for all pairwise subgroups using VCFtools [37]. The Fst measures the real allele frequency differences between the two groups. Fst values range from 0 (i.e., no differentiation) to 1 that represents the complete divergence between the two groups at a given locus. The negative Fst values were converted to zero as there was no biological interpretation of negative values [39]. The Fst values were plotted relative to their physical position within each autosomal chromosome and visualized using the ‘qqman’ package in R 0.1.8 [40]. The top 1% of genome-wide Fst values were considered as the potential selection candidates [21,41].

### 2.5. Cross-Population Extended Haplotype Homozygosity (XP-EHH)

We compared the profiles of EHH between each pair group by calculating XP-EHH statistics using Selscan 2.0.0 software [42] with the max-gap set to 200 kb based on the default program [42]. The XP-EHH statistics can be used to detect selective sweeps in which the selected allele has approached or achieved fixation in one group but remained polymorphic in the other group through comparison of EHH scores of two groups [43]. In the current study, long nap, small pelt size, low fur quality and black mink were considered as control subgroups, which were compared to individuals in the test subgroups including short nap, large pelt size, high fur quality and non-black mink (pastel and stardust), respectively. Finally, the XP-EHH values were normalized by subtracting the mean XP-EHH and dividing by the standard deviation using ‘Norm’ 1.0.3 software [42]. We considered those SNPs with XP-EHH values within the top 1% of positive normalized genome-wide values as selection candidates in each group. Finally, we found the overlapped SNPs located in the top 1% of both Fst and XP-EHH values [21,41]. Gene annotations were then carried out on the 5-kb flanking region around each SNP (5 kbp downstream and upstream of the given SNP).

### 2.6. Nucleotide Diversity (θπ)

Nucleotide diversity was calculated for each group separately using the VCFtools [37]-site-pi option. The θπ ratios were computed as θπ-(long nap, small pelt size, low fur quality)/θπ-(short nap, large pelt size, high fur quality) for pelt quality traits and θπ-(black mink)/θπ-(pastel, and stardust) for coat color traits. For all pairs of groups and were then log2-transformed (log2 (θπ ratios)). Finally, SNPs in the top 1% of log2 (θπ ratios) values were overlapped with the highest 1% of both Fst and XP-EHH values. For each overlapped SNP, a window of the 5-kb flanking region was considered for gene annotations. 

### 2.7. Gene Ontology and Functional Analysis

We used BEDtools 2.30.0 [44] to find the gene IDs overlapped with the candidate regions using general feature format of recent genome assembly of *N. vison* (https://www.ncbi.nlm.nih.gov/genome/16995?genome_assembly_id=1704888; accessed on 15 September, 2022). The biological process, molecular function and cellular component terms were assessed for all genes using PANTHER 14.1 [45]. Benjamini-Hochberg False Discovery Rate (FDR) correction was used for both multiple testing and overrepresentation test. Moreover, Kyoto Encyclopedia of Genes and Genomes (KEGG) pathway analyses [46] was carried out using the g: Profiler [47]. These genes were further investigated by reviewing relevant literatures in relation to the phenotypes or pathways of interest in different groups.

## 3. Results

### 3.1. Selection Signatures Based on XP-EHH and Fst

**Figure 1:**
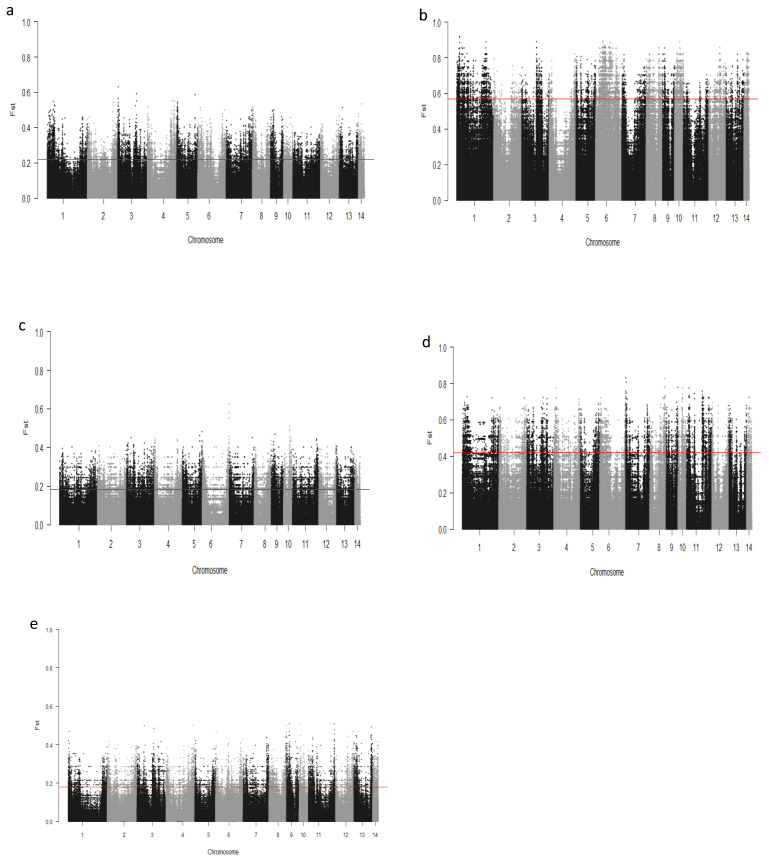
Genome-wide distribution of Fst across chromosomes in different groups of fur quality and coat color in American mink. The horizontal lines indicate the top 1% of values across the entire genome: Nap size (**a**), Overall fur quality (**b**), Skin size (**c**), Pastel_Balck (**d**), and Stardust_Black (**e**).

**Figure 2:**
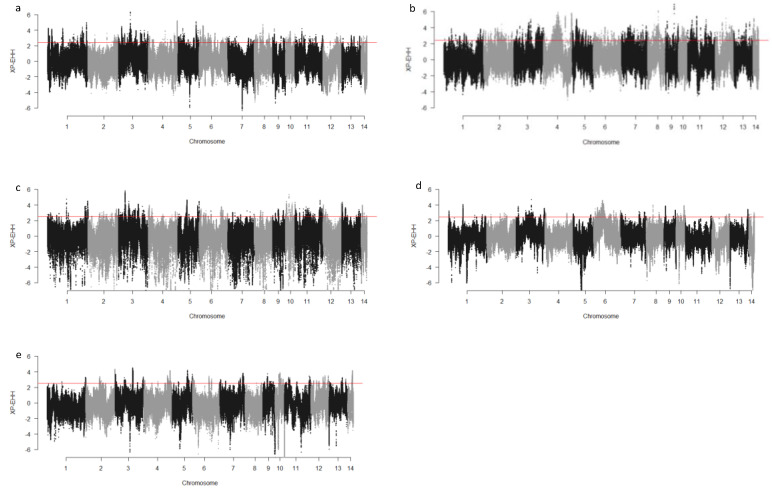
Genome-wide distribution of XP-EHH across chromosome regions in different groups of fur quality and coat color in American mink. The horizontal lines indicate the top 1% of values for each test across the entire genome. High positive values indicate the selection in short nap size (**a**), high overall fur quality (**b**), large skin size (**c**), pastel coat color (**d**), and stardust coat color (**e**).

We computed the Fst values between opposing pairs of each group to investigate the mink genome for potential regions under selection. The distribution of Fst on different chromosomes showing potential signatures of selection in different groups are presented in Figure 1. The differentiation of individuals in each group was also assessed using XP-EHH statistics and its distribution on each chromosome are presented in Figure 2. Supplementary Dataset S1a–e presents the overlaps of top 1% values between Fst and XP-EHH in different groups. There were 4469, 6960, 3880, 5776, and 2804 SNPs with values within the top 1% of both test statistics for Nap size, overall fur quality, pelt size, Pastel_Black, and Stardust_Black groups, respectively. Moreover, a complete list of candidate regions along with their positions was provided in Supplementary Dataset S1a–e. The total number of candidate regions and their associated genes for each phenotypic group are presented in Table 1.

**Figure 3:**
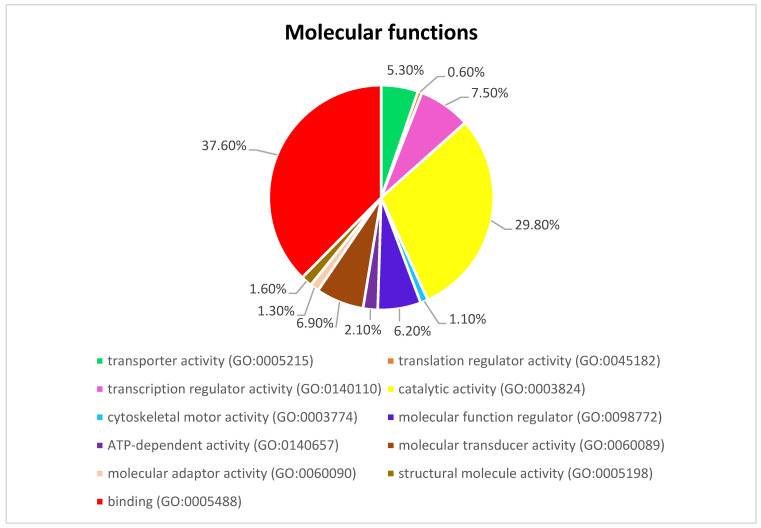
The pie chart of molecular functions attributed to candidate genes detected by overlapping selective signals of Fst and XP-EHH.

**Figure 4:**
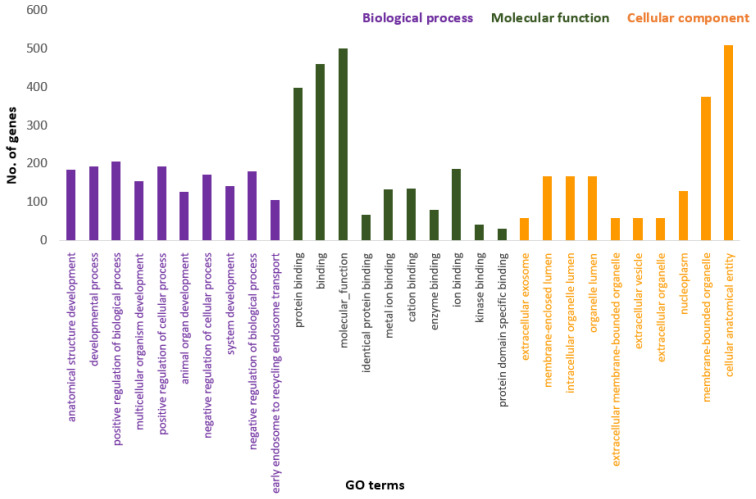
Top ten significant gene ontology terms (GO terms) enriched in overlapping selective signals of Fst and XP-EHH.

Figure 3 presents the Panther pie chart of molecular functions for candidate genes in the putatively selected regions (Supplementary Dataset S3). These results indicated that significant proportions of genes were involved in binding (37.60%), and catalytic activities (29.80%). Based on the overlaps of these tests, 110 genes for nap size, 163 genes for overall fur quality, 98 genes for pelt size, 123 for pastel and 71 for stardust groups were identified to be putatively under selection (Supplementary Dataset S1a–e). The gene ontology analysis resulted in 988, 129, and 261 overrepresented (*p*  <  0.05) GO enrichment terms related to different biological processes, molecular function, and cellular components, respectively (Supplementary Dataset S4a–c). Top ten significant GO terms enriched in candidate regions are presented in Figure 4. In addition, the KEGG pathway analysis revealed two significantly enriched pathways including axon guidance (KEGG:04360) and small cell lung cancer (KEGG:05222) (Supplementary Dataset S4d). Gene ontology revealed the biological roles of several genes related to follicular hair functions including hair cycle process (GO:0022405) and molting cycle process (GO:0022404) *APCDD1, BCL2, TSPEAR, FGFR2,* and *LRP4*; epidermis development (GO:0008544) *HOXB13, SLITRK6, UGCG, COL7A1, FGFR2, MST1, APCDD1, OPN3, LAMB3, LIPK, BCL2,* and *LRP4*; and the Wnt signaling pathway (GO:0016055) *TIAM1, MCC, WNT5B, APCDD1, NR4A2, NLK, PSMB3, CTNND2, RHOA, CCND1, SIAH2, DRAXIN* and *LRP4*. Moreover, we obtained two GO terms with important biological processes related to growth performance of animals including Wnt signaling pathway (GO:0016055) and regulation of striated muscle tissue development (GO:0016202) *SHOX2, MTPN, ACVR1,* and *BCL2*.

### 3.2. Differentiation of Individuals within Each Group Based on θπ Ratios

We used the θπ ratios statistics to put an upper limit to the signatures of selection detected by overlaps of Fst and XP-EHH methods. We filtered top 1% of empirical distribution of log_2_ (θ_π ratios_) in different groups and then only considered the overlapping regions between top 1% of log_2_ (θ_π ratios_) with significant candidate regions identified by previous approaches (XP-EHH and Fst). There were 152, 16, 14, 324, and 7 SNPs within the top 1% of all three methods in nap size, overall fur quality, pelt size, Pastel_Black, and Stardust_Black groups, respectively. Supplementary Dataset S2a–e presents the overlap of top 1% values of all three approaches including Fst, XP-EHH and log_2_ (θ_π ratios_) in different groups. The list of overlapping candidate regions along with the genes involved in those regions are presented in Table 2. The nap size group had the highest number of overlapping regions (12), distributed across the chromosomes 3, 5, 6, and 8.

## 4. Discussion

We reported the first genome-wide analysis of putative signatures of selection for pelt quality and coat color traits using WGS data in American mink. Previous study of signatures of selection in mink was performed using 47,800 SNPs generated by genotyping-by-sequencing (GBS) technique [28], however; the application of high-depth WGS was suggested to improve the accuracy of selective sweep detection [23]. This is because GBS uses the restriction enzymes to reduce the complexity of genome for sequencing; therefore, only a reduced subset of genome is sequenced [48]. Additionally, the current study used the variants called from a chromosome-level reference genome (ASM_NN_V1) which is more comprehensive compared to the previous studies [8,28] that used variants derived from the scaffold-based reference genome [49].

The primary purpose of mink farming is producing a high-quality fur. During the last 100 years of mink farming, ranchers continuously bred mink for more desirable pelt characteristics. The fur’s guard hairs are responsible for its shine and color [50]. Farmers select mink for shorter length of guard hair (nap size) because short-haired furs are more fashionable, while long-haired furs are used for trim [50]. In addition to nap, hair density and healthy and silky appearance of fur influence the price of pelt. We identified 8 key genes (*APCDD1, HOXB13, TSPEAR, TIAM1, OPN3, BCL2, ACVR1,* and *LRP4*) related to hair follicle function, which might play important roles in regulating the guard hair length and density of hair follicles in mink. We obtained several biological terms related to hair growth. The Wnt pathway (GO:0016055) was significant in nap size group, which is the biological pathway considered to be the key regulator of hair follicle morphogenesis [51]. The *APCDD1* gene was detected at chr3: 182,662,938–182,685,570 bp by integrated analysis of Fst and XP-EHH in nap size group. The *APCDD1* product is a membrane-bound glycoprotein that is abundantly expressed in human hair follicles and can interact in vitro with *WNT3A* and *LRP5,* which are the two essential components of Wnt signaling to regulate the hair growth [52]. *HOXB13* (chr5: 46,761,410–46,773,568 bp, nap size group) is involved in the regulation of human hair keratin gene expression. *HOXB13* is a member of the *HOX* multigene family that has an important role in regulation of fetal hair formation [53]. *TSPEAR* gene (chr6: 1,764,452–1,774,452 bp in stardust group) plays a critical role in human hair follicle morphogenesis through regulation of the Notch signaling pathway. It was shown that silencing *TSPEAR* in mouse hair follicles caused apoptosis in hair follicular epithelial cells, leading to a decline in hair bulb diameter [54]. *LRP4* (chr7: 189,857,928–189,874,416, stardust group) mutation can cause defects in hair follicle development [55]. *TIAM1* (chr6: 13,118,844–13,264,781 bp, nap size group) was identified to be essential regulator gene in keratinocytes. The phenotype of keratinocytes with a targeted inactivation of the *TIAM1* gene can cause severe defects in hair follicle morphogenesis, including greatly reduced follicle numbers, failure to progress beyond very early developmental stages, and pronounced defects in follicular keratinocyte proliferation [56]. *OPN3* (chr10: 38,151,971–38,202,287 bp in stardust group) was detected in anagen hair follicles and it was shown that blue light (453 nm), which corresponded to the absorption spectra of OPN3, prolonged the anagen hair growth phase [57]. In the current study, *BCL2* gene (chr3: 136,814,007–136,824,892 bp in pastel group) enriched in GO term related to pigmentation. This gene was shown to be related to normal function of the melanocyte stem cell. *BCL2* null mice displayed the loss of pigmentation after entering the first hair cycle [58]. Moreover, we found *RAB27B* on chr3: 143,746,006–143,797,790 bp with top XP-EHH values of 2.74 and top Fst value of 0.48. *RAB27B* is a small GTPase that shows 71% homology to *RAB27A,* which is involved in melanosome transport and biogenesis. Deletion of *RAB27A* is associated with Griscelli-Pruniéras syndrome type II in human with an unusual silvery-grey hypopigmented color of hair [59]. Evidence suggested that up-regulation of *RAB27B* in melanocytes of the Griscelli-Pruniéras patient can partially acquire the function of *RAB27A*, which can cause an evenly pigmented hair in the absence of *RAB27A* [59].

In addition, selection for larger pelt size and body size is one of the top priorities for mink breeders and has been a key target during mink farming and breeding. We found several genes related to body growth e.g., *NR4A2, ACVR1, RB1, POPDC2, FGFR2, TBX5, and TBX3. NR4A2* (chr3: 54,184,456–54,201,558 bp, in pelt size group) is a member of orphan *NR4A* subgroup, that is involved in the regulation of metabolic function and energy homeostasis [60,61]. Mutations in *ACVR1* are associated with fibrodysplasia ossificans progressive (i.e., abnormal formation of bone in areas of the body such as the ligaments, tendons, and skeletal muscles) [62]. In addition, *ACVR1* was identified as a candidate gene for growth traits in Chinese beef cattle [63]. *RB1* is essential for skeletal myogenesis and development and has an important role in muscular hypertrophy [64,65]. *POPDC2* has an important role in skeletal muscle development, and knockdown of this gene resulted in abnormal development of skeletal muscle [66]. FGFR2 is a member of fibroblast growth factors family and is the most commonly distributed growth factor receptors in mammalian species. It has been demonstrated that FGFR2 is important component of miR-327–FGF10–FGFR2-mediated autocrine signaling mechanism that is involved in control of adipocytes metabolism [67]. In human methylation of *FGFR2* gene was associated with high birth weight centile [68]. *TBX5* and *TBX3* are required for formation and normal development of forelimbs; mutation in these genes is associated with Holt-Oram syndrome [69].

In the present study, we implemented three complementary tests (Fst, XP-EHH and θπ) to identify the candidate regions of positive selection of pelt quality and coat color in American mink. Interestingly, *APCDD1* in nap size group with important function in hair follicles was validated by all three methods, indicating that it can be considered as a reliable candidate of selective sweeps in American mink. Moreover, we identified *BRINP1* gene in the pastel group (chr9: 14,771,596–14,857,094 bp). Previous study on human hair indicated that *BRINP1* was associated with hair loss and hair greying phenotype in human [70]. Another gene was *EPHA6* on chr6: 45,162,190–45,182,915 bp in pastel group. Down-regulation of *EPHA6* expression was associated with low wool density in rabbit [71]. *EPHA3* gene is a member of ephrins which was suggested to act as a hair development promoter [72] and had a potential role in the wool structure of sheep [73].

Selection for pelt quality and body size in mink is certainly a feasible approach to increase the profitability of the mink farms [30]. Genomic selection can be applied as a useful breeding strategy to improve the economically important traits in the mink industry [74]. In this study, numerous loci were detected for pelt quality, pelt size and coat color. Incorporating these loci into current 62 K SNP-chip for mink can be used to improve increase in the accuracy of prediction of genomic estimated breeding values for these traits.

## 5. Conclusions

This study was the first scan for signatures of putative selection for pelt quality and coat color in American mink genome using WGS data. Our results demonstrated that mink genome contained multiple regions likely subjected to selection, some of which appeared to be related to pelt quality, coat color and also body size traits. One strongly selected gene was detected for nap size (*APCDD1*) which was related to hair follicular process. However, more investigation might be required to confirm the roles of these genes in controlling hair follicles in American mink. These results provide a foundation to study the genetic diversity driven by domestication and selection mechanisms in American mink.

## Figures and Tables

**Table 1:** Number of candidate regions and genes detected by overlapping Fst and XP-EHH in differential phenotypic groups of fur quality and coat color in American mink.

Group	Number of Candidate Regions	Number of Genes
Nap size	177	110
Overall fur quality	261	163
Pelt size	204	98
Pastel_Black	201	123
Stardust_Black	103	71

**Table 2:** Overlapping candidate regions and annotated genes identified by three methods (θπ ratios, Fst, and XP-EHH) for fur quality and coat color in American mink.

Chromosome	Position (bp)	Group	Genes
3	182,130,062-182,140,062	Nap size	*RAB31*
3	182,662,938-182,672,938	Nap size	*APCDD1*
3	182,736,504-183,045,067	Nap size	*NAPG, PIEZO2*
3	211,123,039-211,140,367	Nap size	*LDLRAD4*
5	2,940,988-2,952,647	Nap size	*RBFOX3*
5	7,488,192-7,513,312	Nap size	*CDC42EP4, SDK2*
5	7,547,720-7,557,720	Nap size	*CPSF4L, C5H17orf80*
6	27,565,214-27,575,214	Nap size	*USP25*
6	29,333,509-30,616,460	Nap size	*ROBO2*
8	26,111,849-26,121,849	Nap size	*CCM2L*
5	28,909,887-28,931,171	Fur quality	*TMEM199, SARM1*
5	28,921,171-28,931,171	Fur quality	*POLDIP2*
5	29,000,969-29,010,969	Fur quality	*NLK*
3	2,371,161-2,435,977	Pelt size	*RPS6KA2*
3	54,202,833-54,212,833	Pelt size	*GPD2*
4	4,730,111-4,750,052	Pelt size	*FAM135B*
3	127,226,629-127,236,629	Pastel_Black	*ZADH2, TSHZ1*
3	127,816,455-127,839,967	Pastel_Black	*CNDP1, CNDP2*
6	27,436,943-27,589,421	Pastel_Black	*USP25*
6	32,412,680-32,782,053	Pastel_Black	*ROBO1*
6	41,725,705-41,738,492	Pastel_Black	*EPHA3*
6	45,162,190-45,182,915	Pastel_Black	*EPHA6*
6	47,936,381-47,946,381	Pastel_Black	*COL8A1*
9	14,771,596-14,857,094	Pastel_Black	*BRINP1*
10	62,452,956-62,473,337	Pastel_Black	*KCNH1*
7	5,300,435-5,439,094	Stardust_Black	*CDH13*
10	37,647,562-37,657,562	Stardust_Black	*RGS7*

## Data Availability

The datasets used in this work are available from the corresponding author on academic request.

## Supplementary Materials

The following supporting information can be downloaded at: https://www.mdpi.com/article/10.3390/genes13111939/s1, Dataset S1a–e: List of the overlaps of top 1% values of Fst and XP-EHH in different groups and complete list of the candidate regions along with their positions for fur quality and coat color traits in American mink; Dataset S2a–e: List of the overlaps of top 1% values of SNPs for all three approaches including Fst, XP-EHH and log2 (θπ ratios) for fur quality and coat color traits in American mink; Dataset S3: Panther chart molecular functions for candidate genes for signatures of selection of fur quality and coat color in American mink; Dataset S4a–d: List of GO enrichment terms related to biological processes for candidate genes.
